# Biotransformation of protein-rich waste by *Yarrowia lipolytica* IPS21 to high-value products—amino acid supernatants

**DOI:** 10.1128/spectrum.02749-23

**Published:** 2023-09-14

**Authors:** Dorota Wieczorek, Dorota Gendaszewska, Katarzyna Miśkiewicz, Anna Słubik, Katarzyna Ławińska

**Affiliations:** 1 Łukasiewicz Research Network - Lodz Institute of Technology, Lodz, Poland; University of Minnesota Twin Cities, St. Paul, Minnesota, USA

**Keywords:** *Yarrowia lipolytica*, rich-protein waste, biodegradation, bioconversion, amino acids, the activity of enzymes, fertilizers

## Abstract

The yeast strain *Yarrowia lipolytica* IPS 21 was tested for its ability to degrade potentially toxic chrome-tanned leather shavings (CTLS) in a liquid environment. Biological and chemical parameters were monitored during a 48-h period of biotransformation of the protein-rich waste. CTLS was added at a concentration of 0.1–4% (wt/wt) to a modified YPG medium (15 g L^−1^ yeast extract and 5 g L^−1^ NaCl). Biodegradation and bioconversion were performed in a one-step process. It was found that the higher degradation rate depended on the activity of the proteases and the pH of the medium, but not on the initial inoculum ratio and the activity of the dehydrogenase. The highest efficiency of the process was obtained for 4% (wt/wt) CTLS on day 2 (degradation rate 58–67%, biomass production 2.11–2.20 g L^−1^, protease activity 312 U mg^−1^ protein, and pH 9.20). Our results showed that total chromium was probably not transported across the cytoplasmic membrane of *Y. lipolytica* IPS21 and that chromium (III) was not oxidized to chromium (VI). The phytotoxicity of selected amino acid supernatants [2.5% (vol/vol)] was tested after the bioconversion process. It was found that the supernatants had a stimulating effect on the plants tested. The root elongation was 29–28% higher than that of the reference samples. This result makes *Y. lipolytica* IPS21 a potential candidate for safely converting potentially toxic protein-rich wastes into valuable products without enzyme isolation, e.g., amino acid fertilizers.

**IMPORTANCE:**

Enzyme technologies have the greatest practical relevance to environmental trends. Overcoming the barrier of the high cost of carbon substrates used for biotransformation is the main challenge of these methods. The huge potential of the use of extracellular proteases of *Yarrowia* species or amino acids in various industries indicates the need for the extension of basic research on waste as a carbon source for this yeast. The experiments demonstrated that it is possible to use *Y. lipolytica* IPS21 for bioconversion of chrome-tanned leather shavings (CTLS) in a single-step process and to produce high-value amino acid supernatant without having an isolated enzyme. In our study, we show the effect of 2.5% (vol/vol) CTLS supernatant obtained from *Y. lipolytica* IPS21 on the elongation of the root system of selected plants and provide information on the effect of environmental factors on the efficiency of the bioconversion and the migration of chromium.

## INTRODUCTION

The leather tanning industry and the sectors related to it are involved in economic progress. However, waste from tanneries pollutes the air, soil, and water. This causes major health problems. The leather industry generates more than 500,000 tons of solid waste per year (1). The environmental impact of tanneries includes gaseous, solid, and liquid wastes. The modification of raw materials accounts for most of the water, chemicals, and energy. In general, the industrial tanning process is based on chromium (III) salts. Oxidation can be the result of improper storage or physicochemical factors. The oxidative forms (Cr VI) can be hazardous to humans and the environment. Studies have been carried out on the recycling of leather waste (2, 3). Uncontaminated or slightly contaminated chemical fractions of the waste can be used for the production of gelatine, sausage casings, threads, sponges, imitation leather, adhesives, cosmetics, and technical fats. The management of wastes generated after the tanning process is a much more difficult issue. Waste from the chrome tanning process includes splinters, trimmings, and chrome-tanned leather shavings (CTLS) (4). CTLS are leather processing wastes with a very low bulk density. They contain chromium (at least 4%) in their composition and a large amount of collagen (at least 90%). They are a valuable source of protein for raw materials (5). New methods for the development of tannery wastes are in three directions: biodegradation (6), methods for the recovery of protein or other valuable products (4, 7), or the removal and recovery of heavy metals (8, 9). Chrome-tanned leather waste has been the subject of several studies. The aim has been to biodegrade them (10). Batch culture, enzyme extraction, and biotransformation of wastes have shown that only a few microorganisms are capable of degrading CTLS in a three-step process. This group of alkaline protease-producing bacteria and fungi is important in the degradation of chrome tannery wastes (11): *Bacillus subtilis* (12), *B. subtilis* P13 (13), *Bacillus amyloliquefaciens* TCCC 11319, *Penicillium* spp. (10), *Alcaligenes faecalis* (14), *Escherichia coli* (15), or extracellular gelatinases and keratinases of *Bacillus* spp. (16). Other microorganisms capable of producing alkaline, neutral, or acidic proteolytic enzymes include yeasts of the species *Yarrowia lipolytica*, *Candida lipolytica*, and *Aureobasidium pullulans* (17, 18). Reports of CTLS biodegradation and biotransformation to amino acid supernatant in a one-step process by *Yarrowia* species and without enzyme extraction have not been described. The importance of amino acids in agriculture is very high. The main effect of products containing amino acids is to stimulate plant growth and development by providing amino acids ready for incorporation into the metabolic pathway as complete “building blocks.” This has a direct beneficial effect on protein and carbohydrate synthesis and improves the utilization of nitrogen supplied in organic form (19). The study of efficient protease-producing yeast and cell conditions during the one-step bioconversion of chromium-protein-rich wastes can be used to design new and environmentally friendly waste management strategies.

The choice of strategy for biodegradation or recovery of hydrolyzed high-value products for CTLS is extremely important and must include an understanding of the key subcellular mechanisms. The potentially toxic effect of CTLS containing chromium (III) on the ability of yeast to produce enzymes during degradation protein processes is also poorly understood (20). Chromium, cadmium, or other metals are known to be potent inhibitors of microbial activity during biological processes (11). The toxic effect of heavy metals on microorganisms is mediated, for example, by inhibition of enzyme activity or by alterations in protein synthesis and ATP production (21). There are practically non-existent data that show the levels of Cr (III) or Cr (VI) after the chemical or enzymatic hydrolysis of the wastes (22, 23).

In this work, we have tested the ability of *Y. lipolytica* IPS21 to bioconvert different concentrations [0.1–4% (wt/wt)] of CTLS waste in a single-step process. Changes in dehydrogenase and protease activity, medium pH, degradation rate and biomass production, amino acid rate in the medium, and the total chromium and chromium (VI) content in supernatant and residues (biomass and CTLS residues) were determined. The stimulating effect of the supernatants (rich in amino acids) obtained after bioconversion on the plants was also tested using the Phytotoxkit microbiotest. Total nitrogen was measured.

## RESULTS

The current study investigated the one-step degradation and bioconversion of pre-oxidised CTLS waste (sterilized at 121°C) using the *Y. lipolytica* IPS21 yeast strain. This research is the first that uses *Y. lipolytica* IPS21 yeast to bioconvert CTLS in a single-step process and produce high-value amino acid supernatant without having an isolated enzyme. This is the first research that showed chromium migration (yeast biomass/liquid) during the 0.1–1% CTLS’s bioconversion process. This research is the earliest to use a high-quality amino acid supernatant from CTLS bioconversion by *Yarrowia* as a plant stimulant.

### Effect of different CTLS concentrations on degradation rate and growth rate

In this study, the influence of different concentrations of CTLS of *Y. lipolytica* IPS21 on biomass production and degradation of rate was estimated. Biomass production by tested yeast depended on the concentration of CTLS (Table 1).

**TABLE 1:** Effect of the culture medium composition on *Y. lipolytica* IPS21 biomass production and degradation of rate (means ± SD at *P* = 0.05 and *n* = 3)^*c*^

Variants	Biomass production (g L^−1^)		Degradation of rate (%), 48 h
	24 h	48 h	
Y^*b*^ 0.4 mL Y 0.8 mL	1.10 ± 0.05 a 1.45 ± 0.02 b	1.65 ± 0.24 a 1.78 ± 0.06 b	– –
0.1% CTLS^*a*^ Y 0.4 mL 0.1% CTLS Y 0.8 mL	1.19 ± 0.10 a 1.65 ± 0.15 c	1.76 ± 0.09 b 2.08 ± 0.09 c	30.56 ± 2.11 a 41.11 ± 0.34 b
1% CTLS Y 0.4 mL 1% CTLS Y 0.8 mL	1.71 ± 0.09 c 1.90 ± 0.08 d	2.24 ± 0.21 d 2.40 ± 0.11 d	74.31 ± 3.22 e 84.45 ± 1.22 f
4% CTLS Y 0.4 mL 4% CTLS Y 0.8 mL	1.84 ± 0.02 d 1.92 ± 0.03 d	2.20 ± 0.01 d 2.14 ± 0.02 d	58.40 ± 2.11 c 67.55 ± 1.02 d

^*a*^ CTLS, chrome-tanned leather shavings, 0.4–0.8 mL inoculum rate.

^*b*^ Y, YPG-modified medium with *Y. lipolytica* IPS21 yeast.

^*c*^ According to the Tukey-Kramer test, values for the different times assigned for the same letter have not been significantly different (*P* < 0.05).

The highest increase in yeast biomass was observed after 48 h, which is equivalent to 2.24–2.40 g L^−1^, when 1% (wt/wt) CTLS was added. Microorganisms effectively utilized waste as a carbon and nitrogen source. The dynamics of biomass production were correlated with a higher concentration of CTLS (Table 1). The highest effectiveness of CTLS degradation rate [67.55% and 58.31% for 4% (wt/wt) of CTLS] was observed after 48 h. More than 1 g of CTLS was incorporated into the biomass in the form of amino acids or used as a carbon source. The degradation rate after 48 h was lower when CTLS was added at 0.1–1%. This study showed that the biodegradation of CTLS by *Y. lipolytica* IPS21 was more efficient when a higher level of CTLS was used.

### Effect of different CTLS concentrations on proteases and dehydrogenase activity

**Fig 1:**
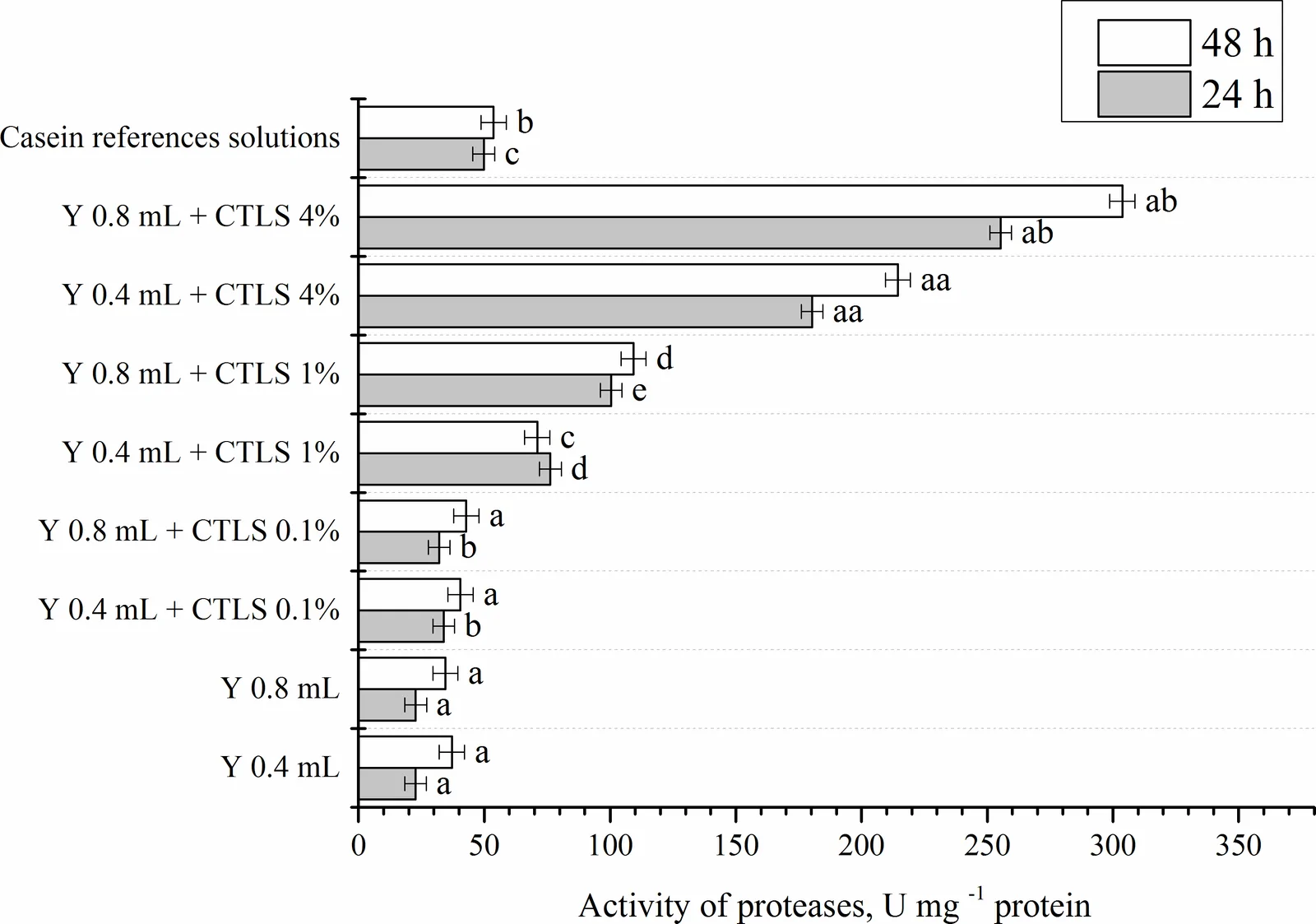
Effect of medium compositions and inoculum rate on the protease’s activity (initial concentration of CTLS was 0.1–4%) during the bioconversion of CTLS by *Y. lipolytica* IPS21 (means ± SD at *P* = 0.05 and *n* = 3). According to the Tukey-Kramer test, values for the different times assigned for the same letter have not been significantly different. Y, YPG-modified medium with *Y. lipolytica* IPS21; CTLS, chrome-tanned leather shavings, 0.4–0.8 mL inoculum rate, 0.1–4% (wt/wt) concentration of CTLS.

Changes in protease activity are shown in Fig. 1. *Y. lipolytica* IPS21 isolates exhibited protease production between 20.11 and 312.4 U mg^−1^ protein whether CTLS was added or not. The highest protease activity was observed at 48 h (312.4 U mg^−1^ protein) when the initial inoculum/concentration CTLS ratio was 2 [0.8 mL and 4% (wt/wt) CTLS]. Other samples had lower protease activity. Interesting results were obtained when casein was added. Proteolytic activity was higher with CTLS as an additional carbon source in some variants [e.g., CTLS 1% (wt/wt) + 0.8 mL], which is 110.12 U mg^−1^ protein than with casein at 52.00 U mg^−1^ protein.

**Fig 2:**
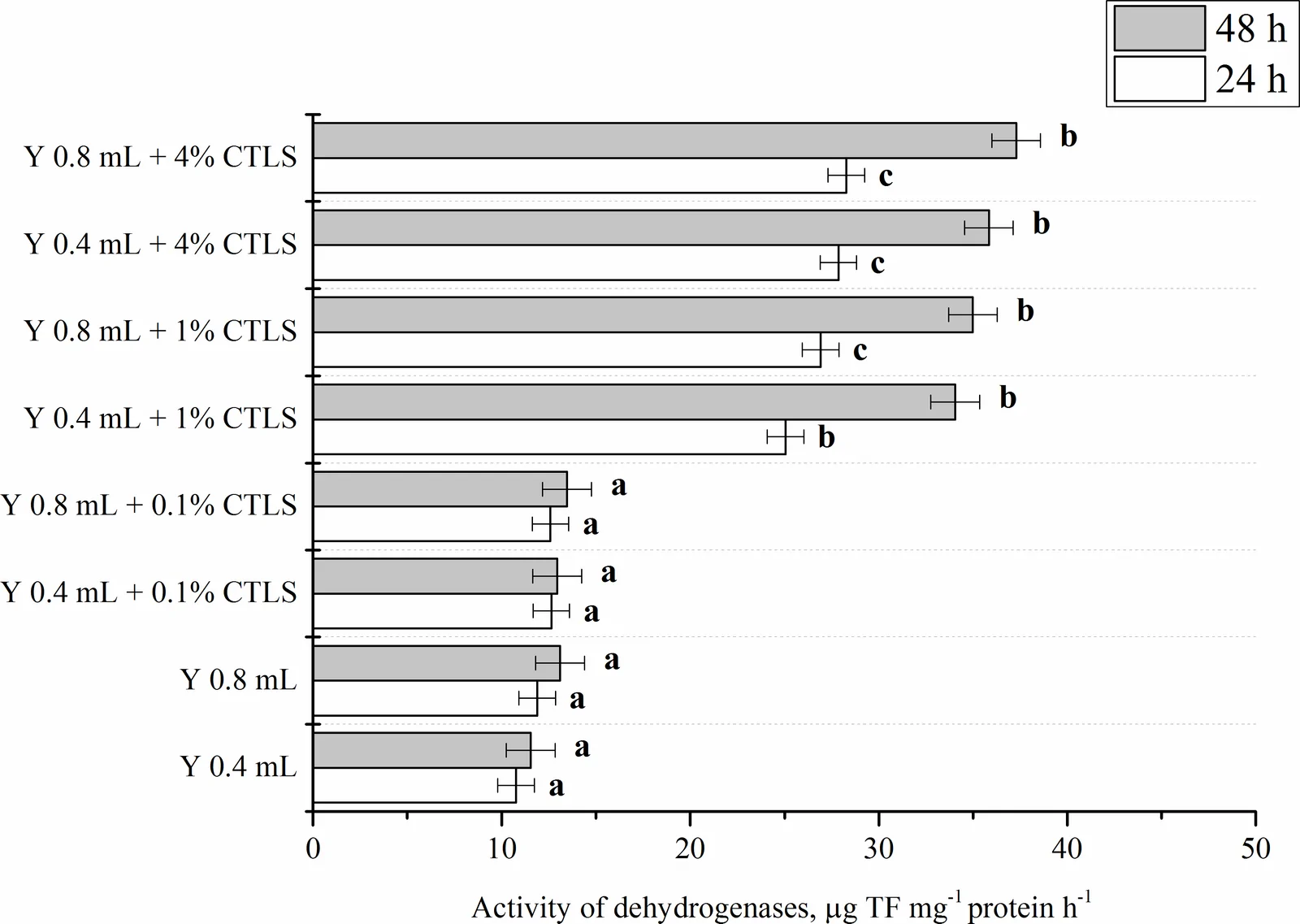
Effect of medium composition and inoculum rate dehydrogenase activity (initial CTLS concentration 0.1–4%) during the bioconversion of CTLS by *Y. lipolytica* IPS21 (means ± SD at *P* = 0.05 and *n* = 3). According to the Tukey-Kramer test, values for the different times assigned for the same letter have not been significantly different. Y, YPG-modified medium with *Y. lipolytica* IPS21; CTLS, chrome-tanned leather shavings, 0.4–0.8 mL inoculum rate, 0.1–4% (wt/wt) concentration CTLS; TF, 1,3,5-triphenyl formazan.

Like the activity of yeast proteases, the activity of dehydrogenases in the first stage (24 h) was lower than in the second stage, from 12.6 to 28.05 µg TF mg^−1^ protein h^−1^ (Fig. 2). The highest values of dehydrogenase activity were observed for 48 h at CTLS concentration of 4% (wt/wt) and 38.04 µg TF mg^−1^ protein h^−1^. Variants with 0.1% or without CTLS showed the lowest values. This activity did not change statistically after the adaptation of *Y. lipolytica* IPS21 to the environmental conditions. Enzyme activity increased during the second stage of bioconversion (48 h), probably as a result of CTLS degradation and related intermediates and biomass production. Significant differences in dehydrogenase activity were observed between samples with and without CTLS at 48 h of the process.

### Effect of different CTLS concentrations on pH

**Fig 3:**
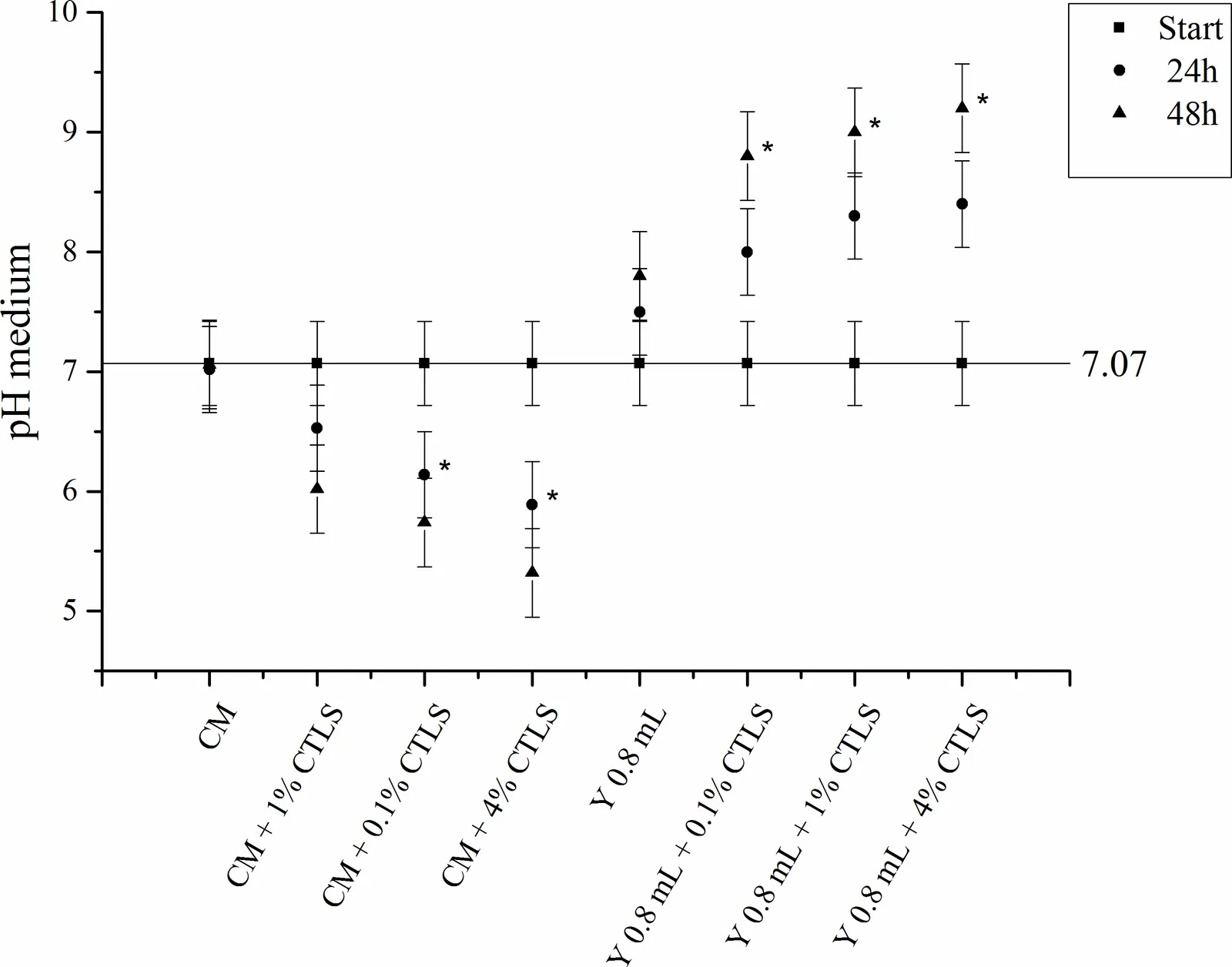
Effect of the culture medium composition on changes in pH with or without *Y. lipolytica* IPS21 (means ± SD, mean 7.21 ± 1.033, *n* = 24). CM, control medium without yeast; Y, *Y. lipolytica* IPS, 0.8 mL inoculum rate, 0.1–4% (wt/wt) concentration of CTLS. Statistically significant differences between sample with *Y. lipolytica* IPS21 and without *Y. lipolytica* IPS (*P < 0.05).

The addition of 0.1–4% (wt/wt) CTLS and *Y. lipolytica* IPS21 to the YPG-modified medium significantly increased the pH within 48 h of cultivation (Fig. 3). The pH was adjusted only at the beginning of the process. It started at 7.07 in all samples. The maximum pH of 9.2 was recorded at hour 48 in variants containing 4% CTLS. The lowest pH value (5.54) was observed after 48 h in the medium with 4% CTLS but without the *Y. lipolytica* IPS21. The decreasing pH value was related to the migration of acidic substances into the medium containing CTLS. The pH value of the aqueous solution of the CTLS was 3.09 (see Materials and Methods).

### Effect of low and high CTLS concentration on amino acid concentration in supernatants

Twenty-nine amino acids and three dipeptides were identified. These included proline-hydroxyproline, hydroxylysine, and glycine-proline (Table in Supplementary Material 1). By comparing the retention time with a standard reference sample, each amino acid was identified. The total amount of identified amino acids in the supernatants of the variants with *Y. lipolytica* IPS21 is shown in Table 2. Higher concentrations of amino acids (7,088.16 mg L^−1^) were obtained at a concentration of 1% (wt/wt) of the CTLS. The lowest overall identified amino acid concentration was found in the control medium (244.57 mg L^−1^) (Table 2). The increase in the concentrations of the selected amino acids may indicate the intensification of the degradation process of the waste or the production of extracellular proteases. The maximum concentration of tyrosine, phenylalanine, lysine, and tryptophan was also observed in the variants with *Y. lipolytica* IPS21 and higher concentrations of CTLS. The concentration of lysine in the variants with *Y. lipolytica* IPS21 was in the range of 1,266.93 mg L^−1^ to 5,134.39 mg L^−1^. In these supernatants, there was six times more lysine than in the absence of yeast (Table in the Supplemental material 1).

**TABLE 2:** The concentration of amino acid in supernatants after the 48 h of bioconversion of CTLS with or without *Y. lipolytica* IPS21 by gas chromatography/mass spectrometry (GC/MS) detection was measured (means ± SD; *n* = 3)^*d*^

	CM^*a*^	CM CTLS^*b*^ 0.1%	CM CTLS 1%	Y^*c*^	Y + CTLS 0.1%	Y + CTLS 1%
	The mean of amino acid concentration (mg L^−1^)					
Aspartic acid^*e*^	26.5± 1.9 a	32.3 ± 4.6 a	105.2 ± 20.5 b	166.4 ± 14.5 c	560.5 ± 19.2 d	511.2 ± 15.1 e
Lysine^*e*^	124.8 ± 17.5 a	212.3 ± 19.5 b	786.6 ± 50.2 c	229.7 ± 22.5 b	1266.9 ± 89.2 d	5134.4 ± 120.3 e
Total without dipeptide	244.6 ± 2.9 a	262.9 ± 5.4 a	1589.6 ± 7.9 c	586.9 ± 4.9 b	2754.4 ± 25.2 d	7088.2 ± 19.0 e

^*a*^ CM, YPG-modified medium.

^*b*^ CTLS, chrome-tanned leather shavings.

^*c*^ Y, YPG-modified medium with *Y. lipolytica* IPS21, 0.1–1% concentration of CTLS.

^*d*^ According to the Tukey-Kramer test, values for the different times assigned for the same letter have not been significantly different.

^*e*^ Other details are in the Supplemental material 1.

### Effect of low and high CTLS concentration on total chromium migration and concentration of chromium (VI)

**Fig 4:**
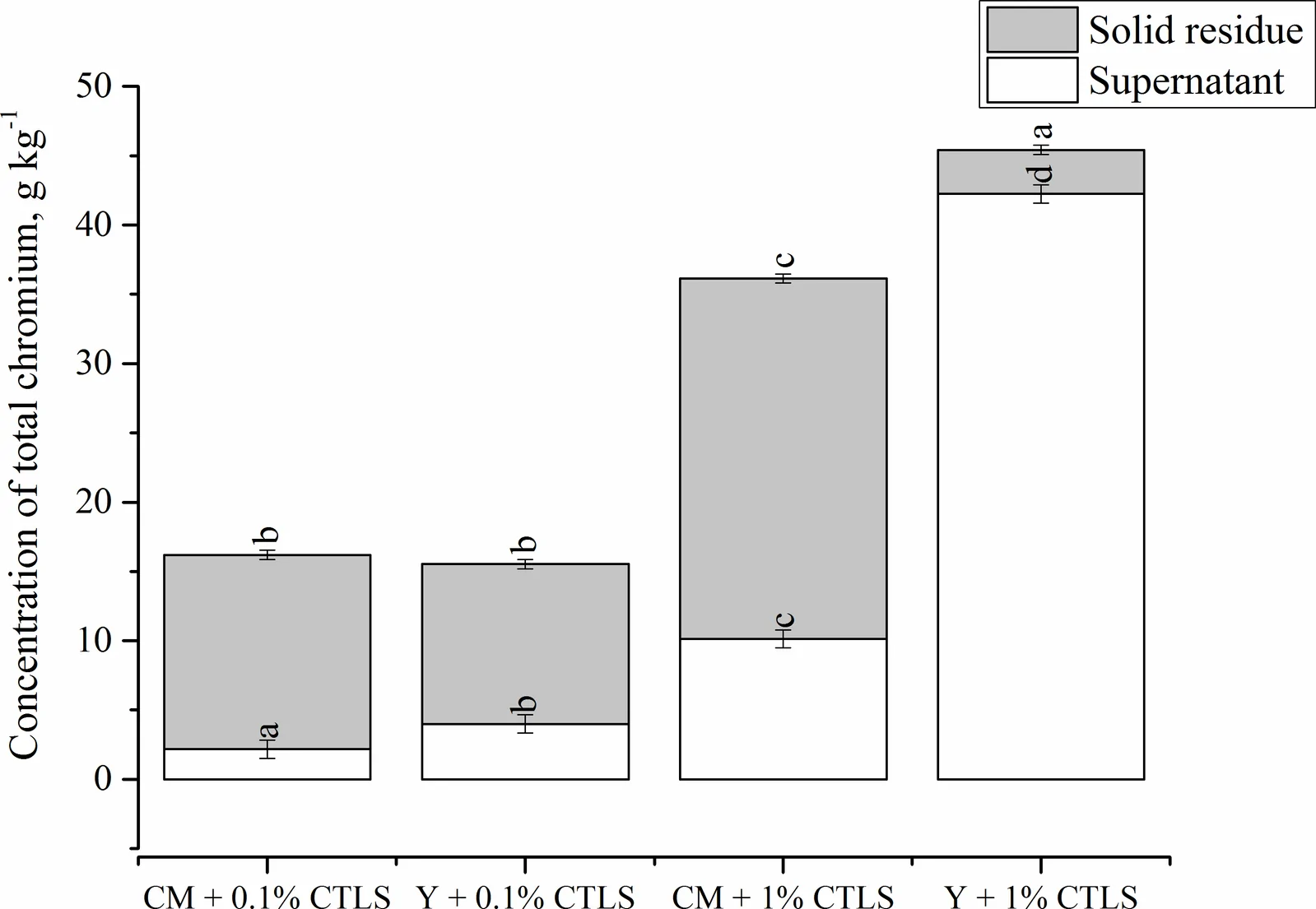
Effect of the culture medium composition on concentration of total chromium with or without *Y. lipolytica* IPS21 (means ± SD at *P* = 0.05 and *n* = 3). According to the Tukey-Kramer test, values for supernatants or residues assigned for the same letter have not been significantly different. CM, YPG-modified medium; Y, YPG-modified medium with *Y. lipolytica* IPS21, 0.1–1% concentration of CTLS.

Total chromium in supernatant and fermentation solid residues (biomass and CTLS residues) was variable (Fig. 4). The results of the 48-h experiments showed that initial waste volume (CTLS) and proteolytic activity of *Y. lipolytica* IPS21 affected total chromium in the liquid phase and biomass rate. The total chromium in liquid phase (supernatants) for a sample containing yeast and 1% CTLS was 42.24 g kg^−1^. As expected, this value was lower for the sample with only 0.1% CTLS (10.31 g kg^−1^). Chromium migrates to the supernatant as a result of the higher bioconversion of CTLS [1% (wt/wt)] by yeast. No chromium (VI) was detected in any of the samples (Table in Supplementary Material 2).

### Effect of supernatants after CTLS bioconversion on inhibition or stimulation plants’ root elongation and amount of nitrogen

**Fig 5:**
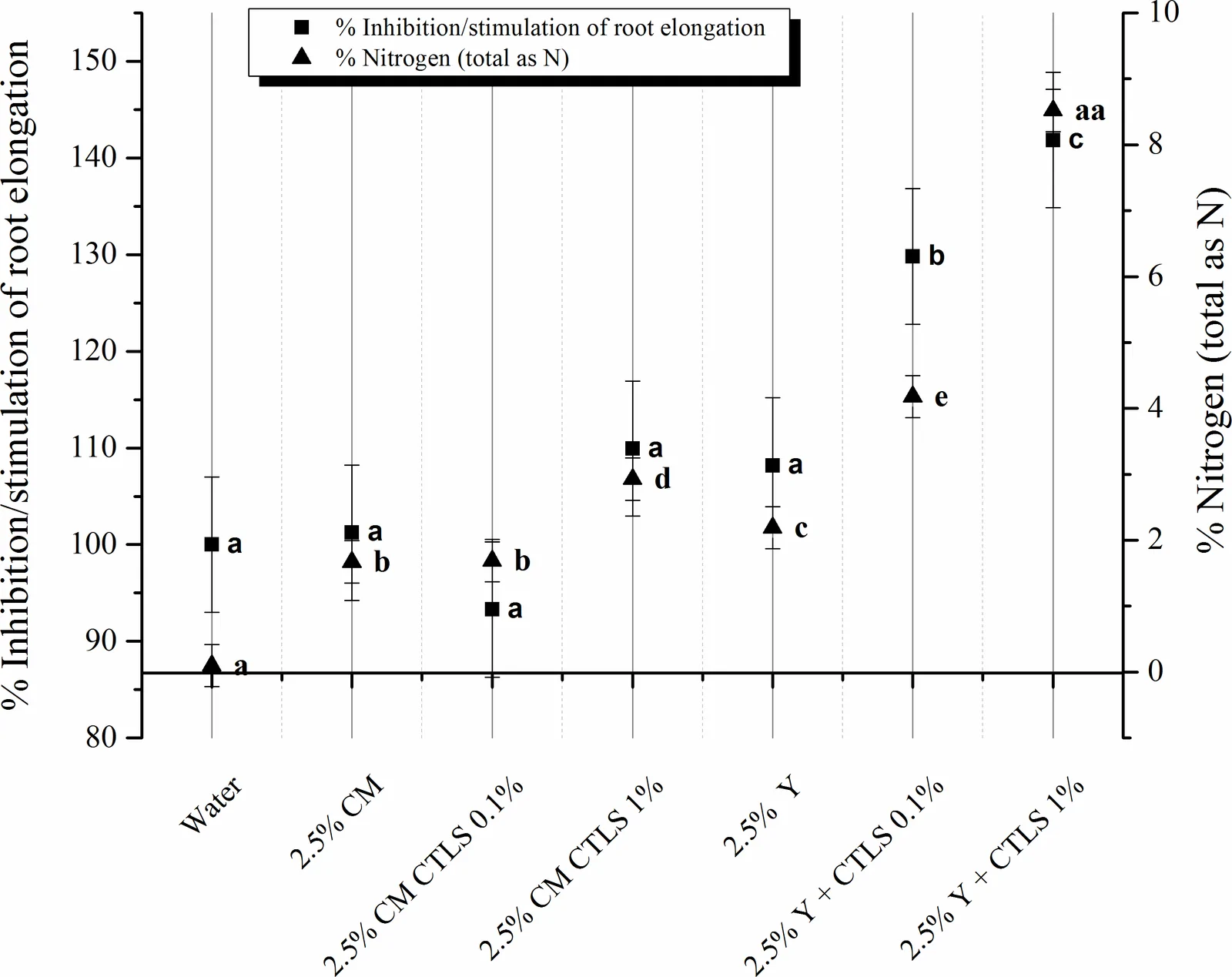
The effect of different of amino acid supernatants [2.5% (vol/vol)] after conversion of waste protein with or without *Y. lipolytica* IPS21 on *Sinapis alba, Lepidium sativum*, and *Sorghum saccharatum* root elongation and percentage of total nitrogen (means ± SD at *P* = 0.05 and *n* = 3). According to the Tukey-Kramer test, values for triangle or square for the same letter have not been significantly different.

The percentages of root elongation and total nitrogen stimulated were high in only two supernatant variants (for bioconversion 0.1–1% CTLS by *Yarrowia*). Root elongation was 29% (*Yarrowia* and 0.1% CTLS) and 38% (*Yarrowia* and 1% CTLS) higher than distilled water (Fig. 5). No statistical differences were observed for the other variants. A high concentration of amino acids in the trials and an increased amount of total nitrogen in these samples were probably responsible for this phenomenon: 4.18% nitrogen (total as N) for 0.1% CTLS or 8.52% nitrogen (total as N) for 1% CTLS with yeast.

## DISCUSSION

*Y. lipolytica* has been described as a microorganism able to assimilate different carbon sources, not only from hydrophilic substrates (glucose, glycerol, organic acids, and alcohol) but also from hydrophobic substrates (fatty acids, triglycerides, esters, and hydrocarbons) (24, 25). *Y. lipolytica* IPS21 was able to grow in the presence of CTLS waste as carbon source (Table 1). The degradation rate (Table 1), protease activity (Fig. 1), and pH of the medium (Fig. 3) were significantly increased by the presence of a higher concentration of CTLS. Many researchers have already described alkaline extracellular proteases as necessary for the effective, economical bioconversion and biodegradation process of protein-rich wastes (26 – 29), which is consistent with these reports. However, our work shows that the costly isolation of enzymes is not required. This is in contrast to previous works (29, 30). The production of bioactive collagen peptides is a desirable goal. These are valuable components. It is important that the fermentation time is kept as short as possible to limit contamination (11). Casein or yeast extract was reported as the main source of nitrogen and carbon toward maximum protease production by yeast (31, 32). In the present study, under the conditions tested, protease production was higher when higher concentrations of CTLS were used as a nitrogen source than in the other cases. The lower protease activity in other variants may be due to insufficient biomass for enzyme production (Table 1). This may be seen in samples containing only YPG-modified medium or low CTLS. Optimum enzyme activity is determined by the appropriate biomass concentration. Higher microbial biomass (Table 1) and thus higher protein production have also been reported in other studies (33). Biological parameters are considered to be sensitive indicators of the degradation or bioconversion of different wastes, such as the activity of proteases and the activity of dehydrogenases (34). Changes in the pH of the medium are also considered as environmental factors during the biosynthesis of proteases. Enzyme activity and nutrient transport into the cell are under the influence of the pH of the culture (31). The intense metabolism, secretion of proteases into the medium, and absorption of organic nitrogen by *Yarrowia* probably caused the significant increase in medium pH (Fig. 3). The decrease in pH was related to the presence of CTLS in the substrate without yeast. The pH of the raw substrate is relatively low. The acidic nature of chrome-tanned leather results from the use of acidic chrome salts [e.g., a chrome alum pH = 3.5; 20°C/chrome B pH (1:10) may be 1.5–2.5] in the wet blue process and the nature of the raw material (35).

In our case, activity of proteases was a more important indicator than dehydrogenase activity. The activity of dehydrogenases differs statistically only in the presence of a higher concentration of waste and between the absence of waste and its low concentration. No changes were observed for 1% and 4% (wt/wt) of CTLS. The changes in dehydrogenase activity can occur as a result of stress caused by the presence of pollutants in environment (34, 36). Dehydrogenase activities were high, indicating that the tested yeasts were in good condition as long as the concentration of waste did not adversely affect yeast growth.

In addition, to the determination of the two enzymatic activities, the concentration of amino acids in the liquid phase was also measured. The sum of all the amino acids was found to be different (Table 2). The increase of selected amino acids in the samples with CTLS and *Y. lipolytica* IPS21 may indicate an intensification of the degradation of waste. Waste can contain up to 90% collagen. High levels of amino acids are found in all CTLS supernatants. A yeast such as *Yarrowia* is characterized by a high content of many exogenous amino acids that build up the biomass (37). Amino acids were also produced as a result of hydrolysis of yeast extract (YPG-modified medium), high protein waste, and secretion of enzymes. The amino acid composition of yeast protein depends mainly on the choice of strain and substrate for its production. It can therefore be largely regulated (33). A high amount of lysine in the culture medium or in the yeast cells is desirable and can be a direction for further work related to the biotransformation of tannery waste by *Yarrowia* sp. into other high-value products such as animal feed (37) or the production of extracellular proteases. The overproduction of extracellular proteases can be used to improve the quality of ripened low-fat cheeses (38) for bioremediation or biosurfactant production (39) too.

In the literature, there are some methods for the production of an amino acid hydrolysate using chrome leather waste. In particular, there is a method for the production of the amino acid fertilizer by hydrolysis of the organic waste using isolated enzymes (40 – 42). In our current work, we sought to determine whether amino acid residues could have toxic or stimulatory properties. Roots were chosen for the stimulation/toxicity analysis because chromium accumulates in roots, which are most exposed to this element. The observed stimulatory effect on root elongation (Fig. 5) of the tested plants was influenced by some supernatant variants (in the 0.1% and 1% CTLS and *Yarrowia* samples) of the supernatant [2.5% (vol/vol)]. In these two treatments, the pH of the supernatant (Fig. 3) was higher than 7.0, and the sum of all amino acids (Table 2) and nitrogen as total N (Fig. 5) was higher than in the control treatments. These three factors may have contributed to the stimulatory effect observed. The chromium III found in the samples did not seem to have a negative effect on the plants. The toxicity of Cr to plants depends on the valence state of Cr: Cr (VI) is highly toxic and mobile, whereas Cr (III) is not toxic at low concentrations (43). Chromium speciation is a determinant of the uptake, transport, and accumulation of the metal in plants (44). Above pH ~5.5, chromium (III) readily forms insoluble hydroxides/oxides. On the other hand, other studies have shown that the presence of higher doses of nitrogen can mitigate the negative effects of too-high chromium doses on plants and even stimulate growth at low chromium (III) levels (45).

### Conclusions

This paper presents the effect of CTLS concentration on biological and chemical parameters during CTLS biotransformation into valuable products by *Y. lipolytica* IPS21. The results of the present study indicate the need for control of biological parameters during productive bioconversion of protein-rich wastes by IPS21 yeast. The extension of knowledge has opened new perspectives in the context of high-quality innovative process design. However, further work is necessary and urgent. Finally, we have confirmed the feasibility of protease expression, amino acid production, and CTLS degradation in a single step. Our research provides an alternative route to bioconversion of protein-rich waste. It does not require the use of pure enzymes. To our knowledge, there have been no studies using supernatants containing amino acids from *Yarrowia* bioconversion of CTLS as plant growth stimulants. Therefore, further studies are needed to investigate the effect of different concentrations of amino acid supernatants with low chromium (III) content on plant growth promotion and to understand the exact mechanism by which they act on different soil types and different crops.

## MATERIALS AND METHODS

### Biological material

*Y. lipolytica* IPS21 from the pure culture of the Łukasiewicz Research Network-Lodz Institute of Technology collection was maintained on “modified YPG” medium: yeast extract 15 g, sodium chloride 2.5 g, agar 25 g per liter of tap water, stocks at 4°C. *Y. lipolytica* strains have a high ability to produce proteases (17, 18). The yeast was isolated (2021) from a soil sample contaminated by petroleum hydrocarbons and high in sulfates, chlorides, phenols, cyanides, and heavy metals. The soil samples were collected in spring from the area of the former “BORUTA” dye factory in Lodz, Poland (51°50′35.4″N 19°23′33.5″E). The purified isolate was subjected to Sanger sequencing and identification at Nexbio in 2022 (Wroclaw, Poland) according to the procedure described in the literature (46, 47). The yeast isolates were identified on the basis of the 18S rRNA gene sequence. The obtained sequence was aligned with nucleotide sequences available in the NCBI database using BLAST (results in Supplementary Material 3a and 3b).

### Waste material (CTLS)

Chrome-tanned leather shavings were obtained after the wet blue tanning process from the tannery in Poland (51°29′37.8″N 21°08′43.1″E). The moisture content of the CTLS used in the process was 15% by weight. The pH of the demineralized aqueous solution (ratio 1:10) was 3.09. The CTLS was characterized by the following parameters: total ash of 8.6% ± 1.4, nitrogen of 16.5% ± 2.2, and chromium (III) oxide of 4.4% ± 0.2 according to the literature (3). The particle size of the CTLS ranged from 1 to 5 mm with an average of 5 mm.

### Verification of potential protease production by *Y. lipolytica* IPS21

*Y. lipolytica* IPS21 was tested for extracellular protease production by plating on skim milk agar plates containing peptone [0.1% (wt/vol)], NaCl [0.5% (wt/vol)], agar [2.0% (wt/vol)], and skim milk [10% (vol/vol)]. The microorganisms were incubated at 30°C for 48 h (31). A change in the color of the medium from milky to clear indicated the presence of extracellular proteases. A protease assay was performed to determine whether they produced acidic, neutral, or alkaline proteases (36). *Y. lipolytica* IPS21 with proteolytic zone is at an average of 14 mm (*n* = 5).

### Determination of potential toxicity of CTLS

The potential toxicity of CTLS to *Y. lipolytica* IPS21 was tested by plating microorganisms on agar-based growth media (potato dextrose agar [PDA]; contents, potato extract [4 g L^-1^], dextrose [20 g L^-1^], and agar [15 g L^-1^]) with the addition of 100 mg CTLS or 300 mg CTLS. The tests were carried out (four replicates) according to the modified PN-EN ISO 20645 standard (48). Samples were placed on plates with agar and CTLS in the central part. Microbiological cultures were grown in Petri dishes and incubated at 30°C for 48 h. After the incubation period, the growth of microorganisms and the zone of inhibition of growth around the working sample were evaluated. No negative effects on *Yarrowia* were observed (Supplementary material 4).

### Biotransformation process

#### Preparation of the inoculum and bioconversion of CTLS to high-value products (amino acid supernatants)

To prepare the *Y. lipolytica* IPS21 inoculum, 10 mL of saline was added to the stock suspension and left for 10 min. After washing the microbial cells from the slopes, the optical density (OD 600) was measured. The final OD 600 is 1,000. The stock suspension (0.4 or 0.8 mL) was added to 100-mL flasks containing 40 mL of sterile modified YPG liquid medium with CTLS of 0.1–4% (wt/wt). This was then incubated for 48 h at 30°C on an orbital shaker (190 rpm). The pH of the medium was adjusted to 7.07 prior to autoclaving at 121°C for 20 min.

#### Determination of CTLS concentration on degradation rate and growth rate

The weight of dry yeast biomass was measured after filtering the cells under pressure on Whatman GC/F filters (100-mm diameter) and washing the cells with acetone and hexane (3:1) and sterile water by the gravimetric method. The samples were dried to constant weight at 80°C. The results are expressed in grams per liter. The degradation rate was calculated from the biomass concentration measurement.

#### Control parameters of the bioconversion process

Proteolytic activity was evaluated according to the literature (49, 50). The reaction mixture contained 200 µL of medium supernatants and 200 µL of 5% azocasein (wt/vol). The azocasein was dissolved in 0.1 M Tris HCL. The samples were then incubated at 40°C for 40 min. To inhibit the reaction, 1% (wt/vol) trichloroacetic acid (TCA) was added. The ratio of TCA to reaction mixture was 3 to 1. After 10 min, the samples were centrifuged (4,000 rpm, 10 min). In the final step, the supernatant was neutralized by the addition of 1,000 µL of 1.0 mol L^−1^ NaOH. The ratio of supernatant to alkaline substance was 1 to 1. Absorbance at 440 nm was measured on a UV/visible spectrophotometer (Rayleigh UV-9200). One unit of proteolytic activity was defined as an increase of 0.01 in 40 min at A440 absorbance.

Dehydrogenase activity was determined according to the literature using 2,3,5-triphenyltetrazolium chloride (TTC) (51). In a first step, the reagents were added to the supernatant diluted twice with TRIS HCl pH 8. First, 1-mL sodium sulfite (3% Na2SO3) was added to 0.8-mL distilled water. Then 0.2 mL of 3% TTC was added. Water was added to the control or reference samples instead of 3% TTC and CTLS supernatant or supernatant with control medium without microorganisms and 3% TTC. The 24-h incubation time was assumed for all tests. The Lowry method was used for protein quantification (52).

The change in pH was measured every 24 h using an Elmetron CP-411 pH meter. Five milliliters of post-culture liquid was taken for measurement.

The determination of amino acids in the supernatants was performed by GC-MS. Samples of 0.1 mL were used for analysis. The samples were analyzed on a 2010 Plus GC chromatograph (Shimadzu, Kyoto, Japan) equipped with the MS GCMS-QP2020 model. The GC column used was ZB-AAA (Phenomenex Inc., Torrance, CA, USA). The temperature programme for the oven: an increase from 110°C to 320°C at a rate of 30°C min^-1^. The column flow was set to He 1.0 mL min^−1^. The injection temperature was set at 300°C. The transfer line to the mass spectrometer was maintained at 200°C. The MS was operated in scan mode (40–450 m/z) and SIM (selected ion monitoring).

#### Determination of the total chromium

The total chromium content in different types of liquid samples and biomass was determined by GFAAS using a Perkin-Elmer AA600 spectrometer (Shelton, CT, USA). Total chromium was determined by GFAAS using a Perkin-Elmer AA600 spectrometer (Shelton, CT, USA). To each sample, 0.1 g or 0.1 mL was added to 7 mL of 65% nitric acid (V). The mineralization process was carried out in three cycles for 20 min at 300°C and 45 bar pressure with maximum microwave power. The sample was then transferred to a 25-mL volumetric flask. Chromium standards (Chem-Lab) with an initial concentration of 1,000 mg L^−1^ were then used. Curve standards were prepared by diluting the stock solution. A buffer was used to eliminate matrix interferences spectrally, e.g., 10% lanthanum (III) nitrate in Pb and Cd analysis.

Determination of chromium (VI) content in residues and supernatants after cultivation was performed in accordance with PN-EN ISO 17075-1:2017–05.

Phytotoxkit liquid sample is a 3-day germination and early growth test with seeds of three higher plants (ISO Standard 18763; Belgium, Ghent). All supernatant [2.5% (vol/vol)] variants were tested in the reference with foam pad, parafilm sheet, white filter paper, black filter paper, and seeds. Each variation was made in three replicates for each plant. Results are expressed as %inhibition or stimulation of root elongation (all data averaged) relative to distilled water. *Sorghum saccharatum* (sorghum, accession no. SOS170622), *Lepidium sativum* (cress, accession no. LES270522), and *Sinapis alba* (mustard, accession no. SIA290322) were used and incubated for 72 h at 25°C (±1°C). The response of the plants to the supernatants was determined by the degree of inhibition or stimulation of seed germination root growth. All measurements were made using the Image J analysis program. The total nitrogen content in each supernatant was determined after the process by the Kjeldahl method (Kjeltec System 1026).

### Data analysis

Mathematical and statistical analyses were performed using Statistica 10.0 (13). Differences were determined by analysis of variance and Tukey-Kramer test, with ±standard errors (SD). The probability level was set at 0.95 for the determination of trees.

## SUPPLEMENTAL MATERIAL

Supplemental file 1 spectrum.02749-23-s0001.pdf The concentration of amino acid

Supplemental file 2 spectrum.02749-23-s0002.pdf Results of chromium(VI)

Supplemental file 3a spectrum.02749-23-s0003.pdf FASTA results

Supplemental file 3b spectrum.02749-23-s0004.pdf Identification of Yarrowia lipolytica

Supplemental file 4 spectrum.02749-23-s0005.pdf Result of potential toxicity

ASM does not own the copyrights to Supplemental Material that may be linked to, or accessed through, an article. The authors have granted ASM a non-exclusive, world-wide license to publish the Supplemental Material files. Please contact the corresponding author directly for reuse.

## References

[1] Onukak I , Mohammed-Dabo I , Ameh A , Okoduwa S , Fasanya O . 2017. Production and characterization of biomass briquettes from tannery solid waste. Recycling 2:17. doi:10.3390/recycling2040017

[2] Colak SM , Zengin G , Özgünay H , Sari Ö , Sarikahya H , Yuceer L . 2005. Utilization of leather industry pre-fleshings in biodiesel production. J Am Leather Chem Assoc 100:137–141.

[3] Ławińska K , Modrzewski R , Serweta W . 2019. Tannery shavings and mineral additives as a basis of new composite materials. Fibres Text East Eur 27:130–139. doi:10.5604/01.3001.0013.2906

[4] Ławińska K , Szufa S , Modrzewski R , Obraniak A , Wężyk T , Rostocki A , Olejnik TP . 2020. Obtaining granules from waste tannery shavings and mineral additives by wet pulp granulation. Molecules 25:5419. doi:10.3390/molecules25225419 33228107 PMC7699417

[5] Quadery AH , MdT U , Chowdhury MJ , Deb AK . 2016. Extraction of polypeptide solution from tannery solid waste (chrome shavings) and its application as Poultry feed. IOSR JAC 9:32–35. doi:10.9790/5736-0911033235

[6] Sanjay MS , Sudarsanam D , Raj GA , Baskar K . 2020. Isolation and identification of chromium reducing bacteria from tannery effluent. J King Saud Univ Sci 32:265–271. doi:10.1016/j.jksus.2018.05.001

[7] Skwarek M , Wala M , Kołodziejek J , Sieczyńska K , Lasoń-Rydel M , Ławińska K , Obraniak A . 2021. Seed coating with biowaste materials and biocides-environment-friendly biostimulation or threat? Agron 11:1034. doi:10.3390/agronomy11061034

[8] Pantazopoulou E , Zouboulis A . 2020. Chromium recovery from tannery sludge and its ash, based on hydrometallurgical methods. Waste Manag Res 38:19–26. doi:10.1177/0734242X19866903 31405339

[9] Pietrelli L , Ferro S , Reverberi AP , Vocciante M . 2020. Removal and recovery of heavy metals from tannery sludge subjected to plasma pyro-gasification process. J Clean Prod 273:123166. doi:10.1016/j.jclepro.2020.123166

[10] Zhang J , Han Z , Teng B , Chen W . 2017. Biodeterioration process of chromium tanned leather with Penicillium sp. Int Biodeterior Biodegradation 116:104–111. doi:10.1016/j.ibiod.2016.10.019

[11] Stefan DS , Dima R , Pantazi M , Ferdes M , Meghea A . 2012. Identifying microorganisms able to perform biodegradation of leather industry waste. Mol Cryst Liq 556:301–308. doi:10.1080/15421406.2012.635983

[12] Aftab NM , Hameed A , ul-Hag I , Run-sheng C . 2006. Biodegradation of leather waste by enzymatic treatment. J Environ Chem Eng 6:462–465.

[13] Pillai P , Archana G . 2012. A novel process for biodegradation and effective utilization of chrome shavings, a solid waste generated in tanneries, using chromium resistant Bacillus subtilis P13. Process Biochem 47:2116–2122. doi:10.1016/j.procbio.2012.07.030

[14] Shanthi C , Banerjee P , Chandra Babu N , Rajakumar G . 2013. Recovery and characterization of protein hydrolysate from chrome shavings by microbial degradation. J Am Leather Chem Assoc 108:231–239.

[15] Akkurt Ş , Oğuz M , Alkan Uçkun A . 2022. Bioreduction and bioremoval of hexavalent chromium by genetically engineered strains (Escherichia coli MT2A and Escherichia coli MT3). World J Microbiol Biotechnol 38:45. doi:10.1007/s11274-022-03235-2 35075546

[16] Mazotto AM , de Melo ACN , Macrae A , Rosado AS , Peixoto R , Cedrola SML , Couri S , Zingali RB , Villa ALV , Rabinovitch L , Chaves JQ , Vermelho AB . 2011. Biodegradation of feather waste by extracellular keratinases and gelatinases from Bacillus spp.. World J Microbiol Biotechnol 27:1355–1365. doi:10.1007/s11274-010-0586-1 25187135

[17] Hernández-Martínez R , Sancho-Solano A , Loera-Corral O , Rojo-Domínguez A , Regalado-González C , Huerta-Ochoa S , Prado-Barragán LA . 2011. Purification and characterization of a thermostable alkaline protease produced by Yarrowia lipolytica. Rev Mex Ing Quim 10:333–341. doi:10.1016/j.procbio.2011.07.013

[18] Bessadok B , Masri M , Breuck T , Sadok S . 2017. Characterization of the crude alkaline extracellular protease of Yarrowia lipolytica YlTun15. J Fish Sci 11:019–024. doi:10.21767/1307-234X.1000137

[19] Rosa R , Hajko L , Franczuk J , Zaniewicz-Bajkowska A , Andrejiová A , Mezeyová I . 2023. Effect of L-tryptophan and L-glutamic acid on carrot yield and its quality. Agron 13:562. doi:10.3390/agronomy13020562

[20] Agustini CB , Neto WL , Priebe G , Costa M , Gutterres M . 2017. Biodegradation of leather solid waste and manipulation of methanogens and chromium-resistant microorganisms. J Am Leather Chem Assoc 112:7–14.

[21] Igiri BE , Okoduwa SIR , Idoko GO , Akabuogu EP , Adeyi AO , Ejiogu IK . 2018. Toxicity and bioremediation of heavy metals contaminated ecosystem from tannery wastewater: a review. J Toxicol 2018:2568038. doi:10.1155/2018/2568038 30363677 PMC6180975

[22] Sole R , Taddei L , Franceschi C , Beghetto V . 2019. Efficient chemo-enzymatic transformation of animal biomass waste for eco-friendly leather production. Molecules 24:2979. doi:10.3390/molecules24162979 31426399 PMC6719968

[23] Ilyas S , Bukhari DA , Rehman A . 2020. Chromium (VI) tolerance and bioaccumulation by Candida tropicalis isolated from textile wastewater. Sustain Environ Res 30:1–8. doi:10.1186/s42834-020-00069-1

[24] Hassanshahian M , Tebyanian H , Cappello S . 2012. Isolation and characterization of two crude oil-degrading yeast strains, Yarrowia lipolytica PG-20 and PG-32, from the Persian Gulf. Mar Pollut Bull 64:1386–1391. doi:10.1016/j.marpolbul.2012.04.020 22622152

[25] Madzak C . 2021. Yarrowia lipolytica strains and their biotechnological applications: how Natural biodiversity and metabolic engineering could contribute to cell factories improvement. J Fungi (Basel) 7:548. doi:10.3390/jof7070548 34356927 PMC8307478

[26] Pokora M , Zambrowicz A , Zabłocka A , Dąbrowska A , Szołtysik M , Babij K , Eckert E , Trziszka T , Chrzanowska J . 2017. The use of serine protease from Yarrowia lipolytica yeast in the production of biopeptides from denatured egg white proteins. Acta Biochim Pol 64:245–253. doi:10.18388/abp.2016_1316 28388696

[27] Yan J , Han B , Gui X , Wang G , Xu L , Yan Y , Madzak C , Pan D , Wang Y , Zha G , Jiao L . 2018. Engineering Yarrowia lipolytica to simultaneously produce lipase and single cell protein from agro-industrial wastes for feed. Sci Rep 8:758. doi:10.1038/s41598-018-19238-9 29335453 PMC5768715

[28] Clerici NJ , Lermen AM , Daroit DJ . 2021. Agro-industrial by-products as substrates for the production of bacterial protease and antioxidant hydrolysates. Biocatal Agric Biotechnol 37:102174. doi:10.1016/j.bcab.2021.102174

[29] Razzaq A , Shamsi S , Ali A , Ali Q , Sajjad M , Malik A , Ashraf M . 2019. Microbial proteases applications. Front Bioeng Biotechnol 7:110. doi:10.3389/fbioe.2019.00110 31263696 PMC6584820

[30] Kolar SL , Ibarra JA , Rivera FE , Mootz JM , Davenport JE , Stevens SM , Horswill AR , Shaw LN . 2013. Extracellular proteases are key mediators of Staphylococcus aureus virulence via the global modulation of virulence-determinant stability. Microbiologyopen 2:18–34. doi:10.1002/mbo3.55 23233325 PMC3584211

[31] Asha B , Palaniswamy M . 2018. Optimization of alkaline protease production by Bacillus cereus FT 1 isolated from soil. J App Pharm Sci 8:119–127. doi:10.7324/JAPS.2018.8219

[32] Suleiman AD , Abdul Rahman N , Mohd Yusof H , Mohd Shariff F , Yasid NA . 2020. Effect of cultural conditions on protease production by a thermophilic Geobacillus thermoglucosidasius SKF4 isolated from Sungai Klah Hot Spring Park, Malaysia. Molecules 25:2609. doi:10.3390/molecules25112609 32512695 PMC7321352

[33] Ahmed S , Mustafa G , Arshad M , Rajoka MI . 2017. Fungal biomass protein production from Trichoderma harzianum using rice polishing. Biomed Res Int 2017:6232793. doi:10.1155/2017/6232793 28367444 PMC5358476

[34] Wieczorek D , Marchut-Mikolajczyk O , Strzelecki B , Gajewska M , Polewczyk A , Antczak T . 2016. The effect of tert-butylhydroquinone (TBHQ) on biodiesel bioremediation in soil samples inoculated with bacterial cells. Int Biodeterior Biodegradation 115:205–211. doi:10.1016/j.ibiod.2016.08.016

[35] Shahriar A , Zohra F-T , Murad A , Ahmed S . 2019. Enhancement of waterproofing properties of finished upper leather produced from Bangladeshi cow hides. JETR 4:63–71. doi:10.24018/ejeng.2019.4.7.1426

[36] Aparna C , Saritha P , Himabindu V , Anjaneyulu Y . 2010. Evaluation of bioremediation effectiveness on sediments contaminated with industrial wastes. Int J Environ Sci 1:607–620.

[37] Juszczyk P , Tomaszewska L , Kita A , Rymowicz W . 2013. Biomass production by novel strains of Yarrowia lipolytica using raw glycerol, derived from biodiesel production. Bioresour Technol 137:124–131. doi:10.1016/j.biortech.2013.03.010 23587815

[38] De Wit M , Osthoff G , Viljoen BC , Hugo A . 2005. A comparative study of lipolysis and proteolysis in Cheddar cheese and yeast-inoculated Cheddar cheeses during ripening. Enzyme and Microbiol Technol 37:606–616. doi:10.1016/j.enzmictec.2005.03.028

[39] Madzak C , Gaillardin C , Beckerich J-M . 2004. Heterologous protein expression and secretion in the non-conventional yeast Yarrowia lipolytica: a review. J Biotechnol 109:63–81. doi:10.1016/j.jbiotec.2003.10.027 15063615

[40] Borges S , Odila J , Voss G , Martins R , Rosa A , Couto JA , Almeida A , Pintado M . 2023. Fish by-products: a source of enzymes to generate circular bioactive hydrolysates. Molecules 28:1155. doi:10.3390/molecules28031155 36770822 PMC9919145

[41] Ławińska K , Lasoń-Rydel M , Gendaszewska D , Grzesiak E , Sieczyńska K , Gaidau C , Epure D-G , Obraniak A . 2019. Coating of seeds with collagen hydrolysates from leather waste. Fibres Text East Eur 27:59–64. doi:10.5604/01.3001.0013.1819

[42] Pecha J , Barinova M , Kolomaznik K , Nguyen TN , Dao AT , Le VT . 2021. Technological-economic optimization of enzymatic hydrolysis used for the processing of chrome-tanned leather waste. Process Saf Environ Prot 152:220–229. doi:10.1016/j.psep.2021.06.009

[43] Sharma A , Kapoor D , Wang J , Shahzad B , Kumar V , Bali AS , Jasrotia S , Zheng B , Yuan H , Yan D . 2020. Chromium bioaccumulation and its impacts on plants: an overview. Plants (Basel) 9:100. doi:10.3390/plants9010100 31941115 PMC7020214

[44] Ertani A , Mietto A , Borin M , Nardi S . 2017. Chromium in agricultural soils and crops: a review. Water Air Soil Pollut 228:190. doi:10.1007/s11270-017-3356-y

[45] Panda SK , Patra HK . 2000. Nitrate and ammonium ions effect on the chromium toxicity in developing wheat seedlings. Proc Natl Acad Sci India Sect B Biol Sci 20:75–80.

[46] Galkiewicz JP , Kellogg CA . 2008. Cross-kingdom amplification using bacteria-specific primers: complications for studies of coral microbial ecology. Appl Environ Microbiol 74:7828–7831. doi:10.1128/AEM.01303-08 18931299 PMC2607152

[47] Gardes M , Bruns TD . 1993. ITS primers with enhanced specificity for basidiomycetes--application to the identification of mycorrhizae and rusts. Mol Ecol 2:113–118. doi:10.1111/j.1365-294x.1993.tb00005.x 8180733

[48] PN – EN ISO 20645: 2006 textile fabrics – determination of antibacterial activity – agar diffusion plate test– polish version.

[49] Coêlho DF , Saturnino TP , Fernandes FF , Mazzola PG , Silveira E , Tambourgi EB . 2016. Azocasein substrate for determination of proteolytic activity: reexamining a traditional method using bromelain samples. Biomed Res Int 2016:8409183. doi:10.1155/2016/8409183 26925415 PMC4748065

[50] Aissaoui N , Marzouki MN , Abidi F . 2017. Purification and biochemical characterization of a novel intestinal protease from Scorpaena notata. Int J Food Prop 2:1–15. doi:10.1080/10942912.2017.1368550

[51] Miksch K . 1985. The Influence of the TTC concentration on the determination of activated sludge activity. Acta Hydrochim Hydrobiol 13:67–73. doi:10.1002/aheh.19850130109

[52] Walker JM . 2009. The Lowry method for protein Quantitation, In The protein protocols Handbook. doi:10.1007/978-1-59745-198-7

