# Supplementary material for: Biotransformation of protein-rich waste by Yarrowia lipolytica IPS21 to high-value products—amino acid supernatants
Source: Microbiol Spectr. 2023 Sep 14;11(5):e02749-23. doi: 10.1128/spectrum.02749-23 (PMC10581069; doi:10.1128/spectrum.02749-23)
Supplement: Supplemental file 1 — The concentration of amino acid [file spectrum.02749-23-s0001.pdf]

The concentration of amino acid in supernatants after the 48 h bioconversion of CTLS with or without *Y. lipolytica IPS21* by GC/MS detection was measured (means ± SD)

	Mean of amino acid concentration, mg L^{-1}					
	CM	CM CTLS 0.1%	CM CTLS 1%	Y	Y + CTLS 0.1%	Y + CTLS 1%
**Alanine**	1.12 ± 0.02	0.50 ± 0.01	7.03 ± 0.08	2.37 ± 0.03	53.9 ± 1.9	20.6 ± 2.4
**Sarcosine**	nd	nd	nd	6.23 ± 0.07	nd	nd
**Glycine**	0.06 ± 0.01	0.04 ± 0.01	0.71 ± 0.01	0.23 ± 0.01	2.40 ± 0.03	2.79 ± 0.02
**Abscisic acid**	1.88 ± 0.04	nd	5.25 ± 0.04	1.42 ± 0.03	nd	21.4± 1.4
**Valine**	0.10 ± 0.01	nd	0.03± 0.01	0.011± 0.001	nd	nd
**Beta aminoisobutyric acid**	0.011 ± 0.001	nd	0.01± 0.01	nd	nd	nd
**Leucine**	nd	nd	nd	0.30± 0.01	nd	nd
**Alloisoleucine**	0.35 ± 0.01	0.07 ± 0.02	2.59 ± 0.24	0.79 ± 0.04	27.6 ± 2.1	24.4 ± 1.1
**Isoleucine**	0.34 ± 0.01	0.09 ± 0.02	3.61 ± 0.08	1.11 ± 0.04	37.8 ± 1.1	57.7 ± 2.1
**Threonine**	0.09 ± 0.01	nd	nd	nd	nd	nd
**Serine**	0.70± 0.01	0.14 ± 0.01	5.18 ± 0.02	1.47 ± 0.05	20.4 ± 1.2	8.08 ± 0.54
**Proline**	0.021± 0.001	0.011 ± 0.001	0.09 ± 0.01	0.90 ± 0.01	156.5 ± 15.2	0.11 ± 0.01
**Asparagine**	1.36 ± 0.04	0.021 ± 0.001	1.62 ± 0.02	0.48 ± 0.01	8.84 ± 0.25	nd
**Thioproline**	0.09 ± 0.01	nd	0.04 ± 0.01	0.011 ± 0.001	0.04 ± 0.01	0.03 ± 0.01
**Aspartic acid**	26.5 ± 1.9	32.3 ± 4.6	105.2 ± 20.5	166.4 ± 14.5	560.5 ± 19.2	511.2 ± 15.1
**Methionine**	14.2 ± 1.2	4.51 ± 0.08	156.6 ± 16.1	41.6 ± 3.1	215.6 ± 21.1	30.8 ± 3.1
**Hydroxyproline**	0.14 ± 0.01	0.01 ± 0.01	0.74 ± 0.03	0.24 ± 0.07	10.5 ± 1.1	11.77 ± 0.04
**Glutamine**	0.28 ± 0.08	0.17 ± 0.07	1.29 ± 0.04	0.34 ± 0.06	nd	3.31 ± 1.08
**Phenylalanine**	5.49 ± 0.05	1.85 ± 0.04	75.5 ± 5.1	19.7 ± 1.1	72.7 ± 2.1	104.5 ± 10.1
**Alpha-amino caprylic acid**	0.25 ± 0.04	0.04 ± 0.01	1.71 ± 0.04	0.58 ± 0.01	30.6 ± 5.1	4.17 ± 0.24
**4-Aminopyrazolonyl Amino Acid**	0.13 ± 0.01	0.04 ± 0.01	0.77 ± 0.03	0.20 ± 0.01	0.09 ± 0.01	1.68 ± 0.04
**Glutamine acid**	8.74 ± 0.65	nd	nd	nd	nd	nd

**Ornithine**	0.90 ± 0.02	0.12 ± 0.03	4.53 ± 0.75	1.21± 0.05	0.92 ± 0.07	3.70± 0.05
**Glycine-Proline (dipeptide)**	0.24 ± 0.01	0.03 ± 0.01	2.41 ± 0.05	0.63 ± 0.02	0.14 ± 0.01	5.62± 0.08
**Lysine**	124.8± 17.5	212.3 ± 19.5	786.6 ± 50.2	229.7 ± 22.5	1266.9 ± 89.2	5134.4±120.3
**Histidine**	1.20 ± 0.05	0.21 ± 0.07	7.48 ± 1.05	2.04 ± 0.05	6.85 ± 0.27	12.61± 2.05
**Hydroxylysine (dipeptide)**	1.11 ± 0.04	0.04 ± 0.01	3.32 ± 0.25	0.91 ± 0.07	24.1 ± 4.1	11.35 ± 0.04
**Tyrosine**	16.7 ± 1.2	3.75 ± 0.23	219.3 ± 25.2	56.6 ± 8.2	129.9 ± 18.6	917.4 ± 60.2
**Proline-hydroxyproline (dipeptide)**	2.25 ± 0.04	0.26 ± 0.01	15.3 ± 3.2	4.64 ± 1.23	237.9 ± 25.1	0.52 ± 0.01
**Tryptophan**	10.7 ± 2.3	1.28 ± 0.04	90.5 ± 8.2	23.5 ± 6.2	53.7 ± 6.9	136.4 ± 12.9
**Cystathionine**	0.24 ± 0.03	0.02 ± 0.01	1.50 ± 0.04	0.42 ± 0.02	14.6 ± 3.5	1.34 ± 0.04
**Cystine**	28.1 ± 4.1	5.43 ± 1.01	111.7 ± 16.2	29.2 ± 6.2	84.0 ± 12.7	109.9 ± 10.1
**Total without dipeptide**	**244.6± 2.9**	**262.9 ± 5.4**	**1589.6 ± 7.9**	**586.9 ± 4.9**	**2754.4 ± 25.2**	**7088.2 ± 19.0**

Note: CM- YPG modified medium, CTLS chrome-tanned leather shavings, Y - YPG modified medium with Yarrowia lipolytica IPS 21, 0.1%-1% concentration of CTLS.
