# Supplementary material for: Biotransformation of protein-rich waste by Yarrowia lipolytica IPS21 to high-value products—amino acid supernatants
Source: Microbiol Spectr. 2023 Sep 14;11(5):e02749-23. doi: 10.1128/spectrum.02749-23 (PMC10581069; doi:10.1128/spectrum.02749-23)
Supplement: Supplemental file 2 — Results of chromium(VI) [file spectrum.02749-23-s0002.pdf]

Results of chromium(VI) determination in residues (biomass and CTLS) and supernatants with or without *Y. lipolytica* IPS21 (means ± SD at p = 0.05, and n = 3).

Leather and Leather-like Materials Testing Laboratory

	Sample	Name of sample	**Chromium (VI)**<br>**PN-EN ISO 17075-1:2017-05**<br>Accredited method
			Chromium(VI) content in (mg L^{-1} or mg $kg^{-1)}$
1	No. 1	**CM (supernatant)**	**Non detected**<br>**≤ 1 mg L^{-1}**
2	No. 2	**CM + 0.1% CTLS (supernatant)**	**Non detected**
3	No. 3	**Y (supernatant)**	**Non detected**
4	No. 4	**Y + 0.1% CTLS (supernatant)**	**Non detected**
5	No. 5	**CM + 1% CTLS (supernatant)**	**Non detected**
6	No. 6	**Y + 1% CTLS (supernatant)**	**Non detected**
7	No. 7	**CM (residues)**	**Non detected**<br>**≤ 1 mg kg^{-1}**
8	No. 8	**CM + 0.1% CTLS (residues)**	**Non detected**
9	No. 9	**Y (residues)**	**Non detected**
10	No. 10	**Y + 0.1% CTLS (residues)**	**Non detected**
11	No. 11	**CM + 1% CTLS (residues)**	**Non detected**
12	No. 12	**Y + 1% CTLS (residues)**	**Non detected**
