# Supplementary material for: Biotransformation of protein-rich waste by Yarrowia lipolytica IPS21 to high-value products—amino acid supernatants
Source: Microbiol Spectr. 2023 Sep 14;11(5):e02749-23. doi: 10.1128/spectrum.02749-23 (PMC10581069; doi:10.1128/spectrum.02749-23)
Supplement: Supplemental file 3a — FASTA results [file spectrum.02749-23-s0003.pdf]

Sequence for three identified strains 1304, 1305, 1306 i FASTA format (Nexbio company).

1304

ACCGGCTTCGGGTGTTACAAACTCTCGTGGTGTGACGGGC

GGTGTGTACAAGACCCGGGAACGTATTCACCGCGGCGTTC

TGATCCGCGATTACTAGCGATTCCGGCTTCATGTAGGCGA

GTTGCAGCCTACAATCCGAACTGAGAATGGCTTTAAGAGA

TTAGCTTGGCCTCACGACTTCGCGACTCGTTGTACCATCC

ATTGTAGCACGTGTGTAGCCCAGGTCATAAGGGGCATGAT

GATTTGACGTCATCCCCACCTTCCTCCGGTTTGTCACCGG

CAGTCTCACTAGAGTGCCCAACTGAATGCTGGCAACTAGT

AATAAGGGTTGCGCTCGTTGCGGGACTTAACCCAACATCT

CACGACACGAGCTGACGACAACCATGCACCACCTGTCACT

TTGTCCCCGAAGGGAAAGCTCGATCTCTCGAGTGGTCAAA

GGATGTCAAGACCTGGTAAGGTTCTTCGCGTTGCTTCGAA

TTAAACCACATGCTCCACCGCTTGTGCGGGTCCCCGTCAA

TTCCTTTGAGTTTCAACCTTGCGGTCGTACTCCCCAGGCG

GAGTGCTTAATGCGTTAGCTGCAGCACTGAAGGGCGGAAA

CCCTCCAACACTTAGCACTCATCGTTTACAGCGTGGACTA

CCAGGGTATCTAATCCTGTTTGCTCCCCACGCTTTCGAGC

CTCAGCGTCAGTTACAGACCAGAGAGTCGCCTTCGCCACT

GGTGTTCCTCCATATATCTACGCATTTCACCGCTACACAT

GGAATTCCACTCTCCTCTTCTGCACTCAAGTTCTCCAGTT

TCCAATGACCCCTCCCCCGGTTGAGCCGGGGGGGCTTTTC

ACATCAGACTTAAAGAACCGCCTGC

GCTCGCTTTACGCCCAATAAATCCGGACAACGCTTGCCAC

CTACGTATTACCGCGGCTGCTGGCACGTAGTTAGCCGTGG

CTTTCTGGTTAGATACCGTCAGGGGATGAGCAGTTACTCT

CATCCTTGTTCTTCTCTAACAACAGAGTTTTACGATCCGA

AAACCTTCTTCACTCACGCGGCATTGCTCCGTCAGACTTT

CGTCCATTGCGGAAGATTCCCTACTGCTGCCTCCCGTAGG

AGTCTGGGCCGTGTCTCAGTCCCAGTGTGGCCGATCACCC

TCTCAGGTCGGCTATGCATCATGGTCTTGGTGAGCCATTA

CCTCACCAACTAACTAATGCACCGCGGGTCCATCCATAAG

TGGTAGCCGAAGCCACCTTTCATCAAGCGACCATGCGGTC

1305

TGCAAGTCGAACGGKGAACACSGTARMTTGCTCTGTGGGATCAGTGGSGAACGGGTGAGT

AACACGCGAGCAACCTGCCCCTGACTCTGGGATAAGCGCTGGAAACGGCGTCTAATACTG

GATATGTGACGTGACCGCATGGTCTGCGTTTGGAAAGATTTTTCGGTTGGGGATGGGCTC

GCGGCCTATCAGCTTGTTGGTGAGGTAATGGCTCACCAAGGCGTCGACGGGTAGCCGGCC

TGAGAGGGTGACCGGCCACACTGGGACTGAGACACGGCCCAGACTCCTACGGGAGGCAGC

AGTGGGGAATATTGCACAATGGGCGGAAGCCTGATGCAGCAACGCCGCGTGAGGGATGAC

GGCCTTCGGGTTGTAAACCTCTTTTAGCAGGGAAGAAGCGAAAGTGACGGTACCTGCAGA

AAAAGCGCCGGCTAACTACGTGCCAGCAGCCGCGGTAATACGTAGGGCGCAAGCGTTATC

CGGAATTATTGGGCGTAAAGAGCTCGTAGGCGGTTTGTCGCGTCTGCTGTGAAATCCCGA

GGCTCAACCTCGGGCCTGCAGTGGGTACGGGCAGACTAGAGTGCGGTAGGGGAGATTGGA

ATTCCTGGTGTAGCGGTGGAATGCGCAGATATCAGGAGGAACACCGATGGCGAAGGCAGA

TCTCTGGGCCGTAACTGACGCTGAGGAGCGAAAGGGTGGGGAGCAAACAGGCTTAGATAC

CCTGGTAGTCCACCCCGTAAACGTTGGGAACTAGTTGTGGGGTCCATTCCACGGATTCCG

TGACGCAGCTAACGCATTAAGTTCCCCGCCTGGGGAGTACGGCCGCAAGGCTAAAACTCA

AAGGAATTGACGGGGACCCGCACAAGCGGCGGAGCATGCGGATTAATTCGATGCAACGCG

AAGAACCTTACCAAGGCTTGACATATACGAGAACGGGCCAGAAATGGTCAACTCTTTGGA

CACTYGTAAACAGGTGGTGCATGGTTGTCGTCAGCTCGTGTCGTGAGATGTTGGGTTAAG

TCCCGCAACGAGCGCAACCCTCGTTCTATGTTGCCAGCACGTMATGGTGGGAACTCATGG

GATACTGCCGGGGTCAACTCGGAGGAAGGTGGGGATGACGTCAAATCATCATGCCCCTTA

TGTCTTGGGCTTCACGCATGCTACAATGGCCGGTACAAAGGGCTGCAATACCGTGAGGTG

GAGCGAATCCCAAAAAGCCGGTMCCAGTTCGGATTGAGGTCTGCAACTCGACCTCATGAA

GTCGGAGTCGCTAGTAATCGCAGATCAGCAACGCTGCGGTGAATACGTTCCCGGGTCTTG

TMCMCWCCGSCCGTCAAGTCATGAAAGTCGGTAACACCTGAAGCCGGTGRCCTAACCCYT

GKGGAGG

1306

AGGAGGTGAAATGACGTAATAATT

GAGAGTTTAAAAAATCCATTTCAAGAAAGCAATGCGATCC

CAGAGGGAACACGCTCCTCCATCCGTGCGGTACGGAATGC

CATACCGCGCAATGTGCGTTCAAAGATTGATGATTCACAT

CTGCAAGTCACAAAAAATATCGCGGTTCGCTGCGTTCTTC

ATCGATGTGAGAGCCAAGAGATCCGTTGTTGATAGTTATA

ATTGAGATAAAATGACGCTGTAATATAATAGAAATCCACA

GAAATAGATAAAATCAATAATGATCCTTCCGCAGGTTCAC

CTACGGAAACCTTGTTACGACTTTTACTTCCTCTAAATGA

CCAAGA
