# Supplementary material for: Biotransformation of protein-rich waste by Yarrowia lipolytica IPS21 to high-value products—amino acid supernatants
Source: Microbiol Spectr. 2023 Sep 14;11(5):e02749-23. doi: 10.1128/spectrum.02749-23 (PMC10581069; doi:10.1128/spectrum.02749-23)
Supplement: Supplemental file 3b — Identification of Yarrowia lipolytica [file spectrum.02749-23-s0004.pdf]

Molecular identification of microorganisms, including 1304, 1305, 1306 strains (Nexbio company).

Nexbio Sp. z o.o.
20-262 Lublin, ul. Dobrzańskiego 3
NIP 9462653876, Regon 361995049
KRS 0000575376

Identyfikacja molekularna mikroorganizmów

RAPORT BADANIA: NR 16/10/2021

**Zleceniodawca**	Dorota Wieczorek, Dorota Gendaszewska SBŁ- Instytut Przemysłu Skórzanego
**Data przyjęcia**	21.10.2021
**Opis próbki**	A, B, C
**Numer laboratoryjny**	**1304, 1305, 1306**
**Pobieranie próbek**	Zleceniodawca
**Okres analizowania**	21.10.2021-28.10.2021
**UWAGI**	

Opis próbki	Numer laboratoryjny	Zidentyfikowany gatunek
A	1304	***Carnobacterium divergens***
B	1305	***Microbacterium paraoxydans/ Microbacterium resistens***
C	1306	***Yarrowia lipolytica***

Sporządził:

mgr Joanna Szczygieł
....
Asystent laboratoryjny

Autoryzował:

...mgr.inż. Jakub Wójcik........
Kierownik Laboratorium

Nexbio Sp. z o.o.
ul. Dobrzańskiego 3, 20-262 Lublin,
tel. + 48 81 820 05 43, +48 534 946 280
e-mail: biuro@nexbio.pl, www.nexbio.pl
