# Supplementary material for: Biotransformation of protein-rich waste by Yarrowia lipolytica IPS21 to high-value products—amino acid supernatants
Source: Microbiol Spectr. 2023 Sep 14;11(5):e02749-23. doi: 10.1128/spectrum.02749-23 (PMC10581069; doi:10.1128/spectrum.02749-23)
Supplement: Supplemental file 4 — Result of potential toxicity [file spectrum.02749-23-s0005.pdf]

Result of potential toxicity of CTLS accordance with the modified PN-EN ISO 20645 standard

100 mg of CTLS	300 mg of CTLS
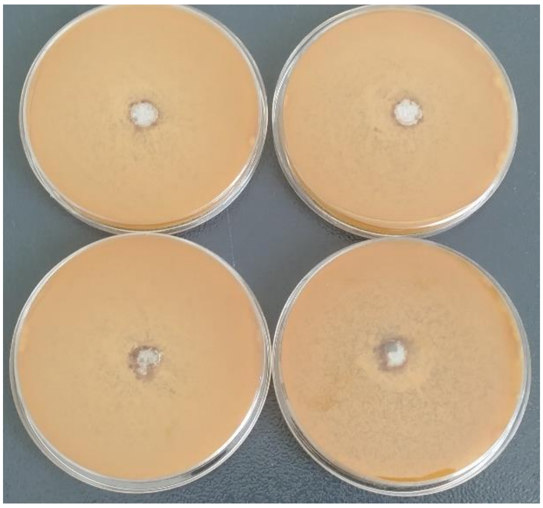	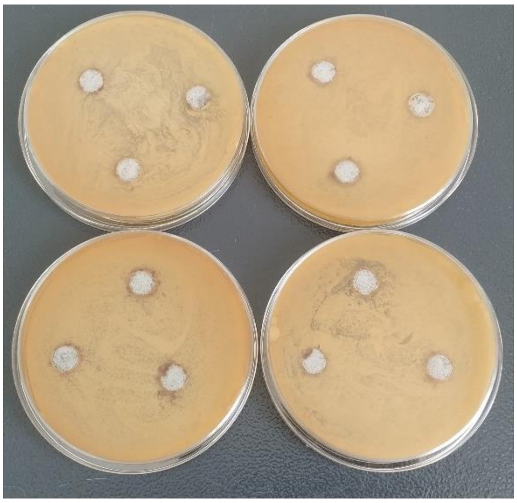
